# 

**Published:** 1872-02-15

**Authors:** 


Alumni Meeting.—The Alumni of the Chicago Medical College will hold their Annual Reunion in the College building, corner of Prairie ave. and 26th street, on Tuesday, March 12th, 1872, at 10 a. m. All Alumni of the College are invited to be present.

MONEY RECEIPTS TO FEB. 15TH, 1872.—H. C. Switcher, $10.00; Williams, $3.00; Henry Ader, $2.00; D. W. Hollister, $3.00; D. H. Patton, $6.00; John Linton, $3.00; E. F. Purdam, $3.00; D. C. Stillions, $3.00; J. A. Booth, $6.00; D. K. A. Steele, $3.00; A. B. Williams, $3.00; Thos. W. Howes, $3.00; J. U. Johns, $4.00; T. H. Blackman, $6.00; V. C. Price, $3.00; E. Ingalls, $3.00; J. Schneck, $3.00; J. C. Kilgore, $3.00; J. R. Webster, $3.00; J. A. Adrain, $3.00; John B. Moore, $3.00; M. O. Heydock, $3.00; A. Hagar, $3.00; T. D. Washburn, $3.00; Jas. Cody, $3.00; E. P. Homer, $3.00; J. Good, $6.00; S. L. Marston, $3.00; J. N. McLean, $3.00; G. W. P. Hodder, $6.00; C. W. Burrell, $3.00; C. B. Reed, $3.00.

